# Novel Approaches to the Diagnosis of Chronic Disorders of Consciousness: Detecting Peripersonal Space by Using Ultrasonics

**DOI:** 10.3389/fneur.2018.00047

**Published:** 2018-02-05

**Authors:** Antonino Naro, Antonino Chillura, Simona Portaro, Alessia Bramanti, Rosaria De Luca, Placido Bramanti, Rocco Salvatore Calabrò

**Affiliations:** ^1^IRCCS centro Neurolesi Bonino-Pulejo, Messina, Italy

**Keywords:** peripersonal space, chronic disorders of consciousness, transcranial ultrasound, motor imagery, cerebral blood flow

## Abstract

The assessment of behavioral responsiveness in patients suffering from chronic disorders of consciousness (DoC), including Unresponsive Wakefulness Syndrome (UWS) and Minimally Conscious State (MCS), is challenging. Even if a patient is unresponsive, he/she may be covertly aware in reason of a cognitive-motor dissociation, i.e., a preservation of cognitive functions despite a solely reflexive behavioral responsiveness. The approach of an external stimulus to the peripersonal space (PPS) modifies some biological measures (e.g., hand-blink reflex amplitude) to the purpose of defensive responses from threats. Such modulation depends on a top-down control of subcortical neural circuits, which can be explored through changes in cerebral blood flow velocity (CBFV), using functional transcranial Doppler (fTCD) and, thus, gaining useful, indirect information on brain connectivity. These data may be used for the DoC differential diagnosis. We evaluated the changes in CBFV by measuring the pulsatility index (PI) in 21 patients with DoC (10 patients with MCS and 11 with UWS) and 25 healthy controls (HC) during a passive movement and motor imagery (MI) task in which the hand of the subject approached and, then, moved away from the subject’s face. In the passive movement task, the PI increased progressively in the HCs when the hand was moved toward the face and, then, it decreased when the hand was removed from the face. The PI increased when the hand was moved toward the face in patients with DoC, but then, it remained high when the hand was removed from the face and up to 30 s after the end of the movement in the patients with MCS (both MCS+ and MCS−) and 1 min in those with UWS, thus differentiating between patients with MCS and UWS. In the MI task, all the HCs, three out of four patients with MCS+, and one out of six patients with MCS− showed an increase–decrease PI change, whereas the remaining patients with MCS and all the patients with UWS showed no PI changes. Even though there is the possibility that our findings will not be replicated in all patients with DoC, we propose fTCD as a rapid and very easy tool to differentiate between patients with MCS and UWS, by identifying residual top-down modulation processes from higher-order cortical areas to sensory-motor integration networks related to the PPS, when using passive movement tasks.

## Introduction

Patients with chronic disorders of consciousness (DoC), including unresponsive wakefulness syndrome (UWS) and minimally conscious state (MCS), show a deterioration of the awareness of self and the environment despite a preserved wakefulness (1, 2). The differential diagnosis between these two entities is essentially based on clinical scales [including the JFK Coma Recovery Scale-Revised (CRS-R)] (3) that focus on the level of behavioral responsiveness to different types of stimuli (4). While patients with UWS disclose no voluntary behavioral responses, individuals with MCS show variable signs of consciousness and are subcategorized into MCS+ and MCS− based on the level of complexity of observed behavioral responses. Specifically, the former shows command following, intelligible verbalization or gestural or “intentional communication,” while the latter only shows minimal levels of behavioral interaction (i.e., non-reflex movements) (5).

Nonetheless, making the distinction between MCS and UWS patients is challenging, as reflected by the high misdiagnosis rate (6). Indeed, the clinical presentations of these two entities can be relatively similar in many cases, although having different levels of awareness, and discriminating between reflexive and willful behavior can be difficult (7). In fact, the clinical assessment can be biased by several sources of false negative results, including abnormalities in brain arousal and attention, sensory and motor output impairment, language comprehension, restraining and immobilizing techniques, and pain (8–10). These aspects can determine clinical conditions that have been labeled as MCS*, cognitive-motor dissociation, Functional Locked-In Syndrome, Vegetative State with hidden consciousness or with preserved islands of consciousness (5, 11–16), in which a behaviorally unresponsive patient is covertly aware, i.e., aware but unable to manifest it (owing to, e.g., a severe motor impairment, with particular regard to the motor cortico-thalamocortical circuits) (17).

Therefore, advanced paraclinical approaches complementing the clinical assessment, including functional neuroimaging and neurophysiology, aimed at demonstrating covert willful behavior (e.g., by looking at task-dependent and task-independent brain activation as compared to that observed in conscious healthy controls) could help in DoC differential diagnosis. About that, the evaluation of the peripersonal space (PPS) may be useful. PPS defines the region of space immediately surrounding the body in which objects can be grasped and manipulated. It has been observed that a stimulus approaching the PPS provokes a more vigorous defensive reaction than a homologous stimulus outside the PPS (18–23). This modulation depends on a cortico-thalamo-brainstem top-down control of the bottom-up information (arousing, in turn, the top-down control) (20, 21). Thus, any difference in behavioral response should be the result of some stimulus becoming salient through a voluntary top-down process (24). At the same time, the assessment of behavior-related brain responses may disclose useful information, albeit indirect, on the degree of deterioration of the cortical-thalamocortical connectivity in patients with DoC and, thus, on the level of awareness.

An easy and quick way to study PPS and the related top-down control may consist of the assessment of its neurovascular function by using functional transcranial doppler (fTCD) sonography. fTCD represents an extension of the standard TCD, allowing to assess the modulation of cerebral hemodynamics during brain activation paradigms, e.g., the execution of motor tasks, motor imagery (MI), and sensory stimulation (25–27). In fact, mental and motor activities augment regional metabolism and modify auto-regulatory mechanisms that, in turn, influence cerebrovascular resistance, thus resulting in an increase in cerebral blood flow velocity (CBFV) (28–32). By measuring CBFV, it is possible to estimate functional connectivity subserving the execution of a motor task, given that cerebral blood flow indirectly assesses the functional connectivity among the hubs constituting brain networks while transferring information across brain regions (33). In fact, cerebral blood flow is proportionate to the functional connectivity strength in a connection–distance dependent fashion and to the level of behavioral performance (33). fTCD has a low spatial resolution and is an easy tool in comparison to more sophisticated devices [including functional magnetic resonance imaging (fMRI) and electroencephalography (EEG)], it is non-invasive, readily available, easily repeatable, and has an excellent temporal resolution (5 ms) to document hemodynamic changes (34).

To the best of our knowledge, no study has yet investigated hemodynamic changes related to PPS perturbation by using fTCD. This study aims to measure the modulation of CBFV related to stimuli approaching the PPS-face; these hemodynamic changes should reflect the level of integrity of the top-down cortical-thalamo-brainstem pathways and, indirectly, the level of awareness.

## Materials and Methods

### Participants

Twenty-two patients with DoC attending our neurorehabilitation units (12 males and 10 females, mean age 52 ± 17 years, range 19–73; MCS: 53 ± 16 years; UWS: 54 ± 18 years; 11 patients were in a UWS—disease duration 9 ± 6 months and 10 in an MCS—disease duration 8 ± 4 months) were consecutively included in the study. The DoC diagnosis was based on the neurobehavioral assessments (performed twice a day for 1 month before the assessment for study eligibility, using the CRS-R) (35) and the available functional studies (neuroimaging and EEG). Only five patients previously underwent advanced analyses, which furnished results in keeping with the clinical diagnosis. Moreover, patients with MCS were categorized into MCS+ and MCS− based on the level of complexity of observed behavioral responses (response to the command, intelligibly verbalization, and intentional communication) (5).

Persons with hemodynamic stenosis of neck vessels (as measured by cervical and TCD ultrasound to exclude hemodynamically significant stenosis in the target territory), cardiovascular/hemodynamic instability, severe spasticity that limits upper limb movements, hypo/hypercapnia, and vasodilatory or vasoconstrictor drug treatment, were excluded from the study. fTCD measurements were compared with 25 healthy control subjects (HC) (11 males and 14 females, mean age 55 ± 15 years, range 25–75), who did not show hemodynamic stenosis of neck vessels and did not take vasodilatory or vasoconstrictor drugs. The clinical and demographic characteristics are shown in Table 1. The level of behavioral responsiveness was assessed using the CRS-R (which was performed twice a day for 1 month before study inclusion by two trained and experienced neurologists). The best CRS-R score observed was used for the analyses. The research followed the principles of the Declaration of Helsinki and was approved by the Ethics Committee of the Institute. HC and the legal surrogates of patients with DoC gave their written informed consent to participate in the study.

**Table 1:** Clinical–demographic characteristics.

	Age (years)	Gender	BI	DD (months)	CRS-R	RF	Treatment
MCS+ (*n* = 4)	63	M	V	6	18	1.7	B, A
	25	M	V	12	17	5.7	B
	65	F	T	16	16	2	AED
	63	M	T	7	16	1.7	B, LD

MCS− (*n* = 6)	53	F	V	6	14	4.7	B, LD
	56	F	T	6	12	None	B, LD, A
	64	M	T	8	14	1.6	LD
	54	M	T	5	11	3.8	AED, A
	62	F	V	7	9	None	LD, A
	20	M	V	7	9	2	B

Mean ± SD	53±	6M	5T	8±	14±		
	16	4F	5V	4	3		

UWS (*n* = 11)	67	M	T	8	6	1	B, LD, AED, A
	60	M	T	6	6	2	LD, A
	19	M	V	5	6	4	B, AED
	65	M	T	7	6	2.6	LD, AED
	53	F	T	18	5	3.7	LD, AED, A
	73	M	V	24	5	4.7	B, LD, AED, A
	20	M	V	4	5	2.4	AED
	65	F	T	7	4	4.7	LD, AED, A
	55	F	T	6	4	5.7	AED
	57	M	T	6	4	None	B, LD
	59	F	V	6	3	2.6	B, A

Mean ± SD	54±	7M	7T	9±	5±		
	18	4F	4V	6	1		

Mean ± SD	54±	7M	7T	9±	5±		
	18	4F	4V	6	1		

*BI, etiology of brain injury; CRS-R, Coma Recovery Scale-Revised; DD, disease duration; F, female; M, male; MCS, Minimally Conscious State; T, traumatic; UWS, Unresponsive Wakefulness Syndrome; V, vascular; RF, risk factors (1. physical inactivity, 2. tobacco, 3. blood lipids, 4. hypertension, 5. obesity, 6. family history, 7. diabetes, 8. coagulopathies); LD, l-DOPA; AED, antiepileptic drugs; A, analgesics; B, baclofen; (+), patients with MCS+*.

### Experimental Procedure

**Figure 1:**
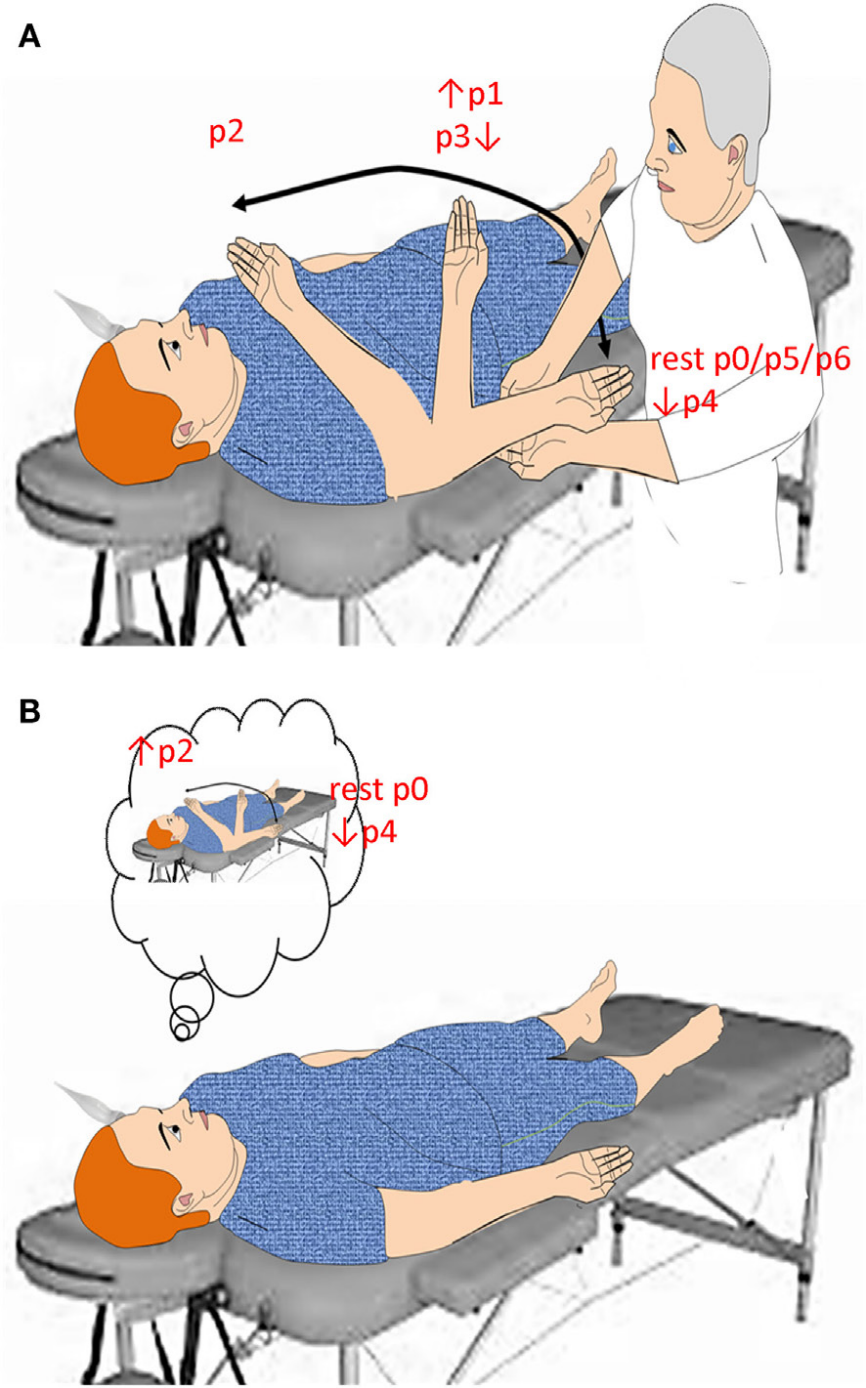
Summarizes the experimental paradigm. Concerning passive movement task **(A)**, the subject was verbally instructed to keep the eyes open, to relax, and to be prepared to be moved the right upper limb from trunk toward the face and vice versa. The speed of passive movement was kept as constant as possible. When the hand reached a predetermined position (p), the examiner kept hold of the participant’s forearm up to the next movement onset, which occurred every 15 heartbeats. Concerning motor imagery task **(B)**, the subject was provided with two sequential instructions, provided every 15 heartbeats: (i) move your right hand toward the face and keep hold of it, and (ii) came back where you started. All instructions were 2 s in length and presented by loudspeakers.

The participant was lying supine on a bed, in a quiet and mild-lighted room, with the eyes open (CRS-R arousal protocol guaranteed this in patients with DoC). The right upper limb was lying along the trunk with the palm facing up, the right arm in a position that allowed the forearm to move toward the eyes (without touching the face) (Figure 1). Blood pressure (from left upper limb) and heart rate (HR) were continuously monitored. The subjects were provided with two motor tasks in random order. In the “passive movement” task, an experimenter mobilized the right upper limb across five different positions, so as to move the hand toward and backward the face, i.e., forearm extended on the arm (p0 = 180°), forearm flexed on the arm at 90° (p1), forearm flexed on the arm at 10° (p2), and then going back to 90° (p3), and 180° (p4) (Figure 1A). The speed of passive movements was kept as constant as possible. Each movement occurred every 15 heartbeats. During each stationary period, the examiner kept hold of the participant’s forearm. This experiment was performed to verify the role of the two afferent pathways (optic and proprioceptive) in the modulation of cerebral hemodynamic responses.

In the “motor imagery” task, all participants were instructed to mentally perform an upper limb flexion movement toward the face (i.e., from p0 to p2) and an extension movement backward the face (i.e., from p2 to p4) (Figure 1B). Each movement occurred every 15 heartbeats. This experiment was conducted to evaluate whether and how MI affects cerebral hemodynamics.

As a control experiment, we performed a condition in which the experimenter’s hand entered the PPS-face, without actual movement of the subject of his or her own body in 10 out of 25 HCs. fTCD was recorded during this task with the same modalities of the “passive movement” task.

By using fTCD, we measured the pulsatility index (PI) from the middle (MCA), anterior (ACA), and posterior (PCA) cerebral artery at rest (p0_rest_) and during the entire motor task. The resting condition (p0_rest_) allowed the establishment of the regulatory parameters for subsequent sessions. We also measured PI from MCA 30 (p5) and 60 s (p6) after the end of the passive movement (p5 and p6 being identical to p0). The main experiments were repeated twice, to control for any variation within the subjects, averaging the results obtained from the two trials. The fTCD recording was performed twice for each intracranial vessel. The head was held in place by a headrest to minimize head movements. All HCs were asked to avoid caffeine, alcohol, and nicotine for 24 h before the measurement, due to these substances well-known effects on vascular reactivity (36).

### Functional Transcranial Doppler

Functional transcranial Doppler was performed by using a conventional color-coded ultrasound system equipped with a 2–5 MHz phased array transducer (iU22 Philips, Healthcare Solutions, Bothell, WA, USA). The examination was performed through the left temporal acoustic bone window and with the transducer placed anteriorly to the tragus and upwards of the zygomatic arch. The fTCD probe was fixed on the head of the participant by using a hard hat to guarantee the same displacement over the measures and conditions. The peak systolic velocity (PSV), end-diastolic velocity, mean velocity (Vm), and PI were measured for each intracranial vessel and were averaged. Age- and gender-corrected PI was calculated, according to the formula (PSV − EVD)/Vm.

### Statistical Analysis

Pulsatility index and HR modulation were analyzed by using an ANOVA with the factors: *hand-position* (six levels for passive movement: p0 → p1, p1 → p2, p2 → p3, p3 → p4, p4 → p5, and p5 → p6; two levels for MI: p0 → p2 and p2 → p4), *task* (two levels: passive movement and MI), and *group* (three levels: HC, MCS, and UWS). We did not aim at assessing the differences between the fTCD of the three main brain vessels as no between-vessel differences have been reported (25, 26). A *p*-value < 0.05 was considered significant. Conditional on a significant *F*-value for the *hand-position* factor, *post hoc t-*tests were performed for each group and motor task (with Bonferroni correction, α = 0.0125). The Greenhouse–Geisser method was applied to compensate for the possible effects of non-sphericity in the compared measurements. All data are presented as mean ± SD or as percent changes, where appropriate.

Given that the patients with vascular brain damage frequently have long-lasting vascular risk factors (such as arterial hypertension), which may affect cerebral hemodynamics and modify the mechanisms of cerebral vascular autoregulation (including the limits within the standard window of autoregulation), brain injury etiology and cardiovascular risk factors were included as covariates in the multivariate analysis. β values [standardized regression coefficients (SRCs)], which is a measure of how strongly each predictor variable influences the criterion (dependent) variable, are provided. The higher the β value, the greater the impact of the predictor variable on the criterion variable. If the β coefficient is equal or nearly to 0, then there is no relationship between the variables.

Clinical-electrophysiological correlations (among CRS-R, PI, and HR) were assessed by using the Spearman correlation test.

In the context of single-subject sensitivity analysis, we employed a linear regression model; SRCs were considered as direct measures of sensitivity. The sensitivity and specificity of the electrophysiological measures employed to distinguish accurately between MCS and UWS were calculated by measuring the area under the curve (AUC) of receiver operating characteristic (ROC). Finally, intraindividual variability between the trials was also calculated in terms of SD of the individual’s scores over the trials, thus reflecting the degree of within-person fluctuation over time. Higher scores reflected greater variability.

## Results

**Figure 2:**
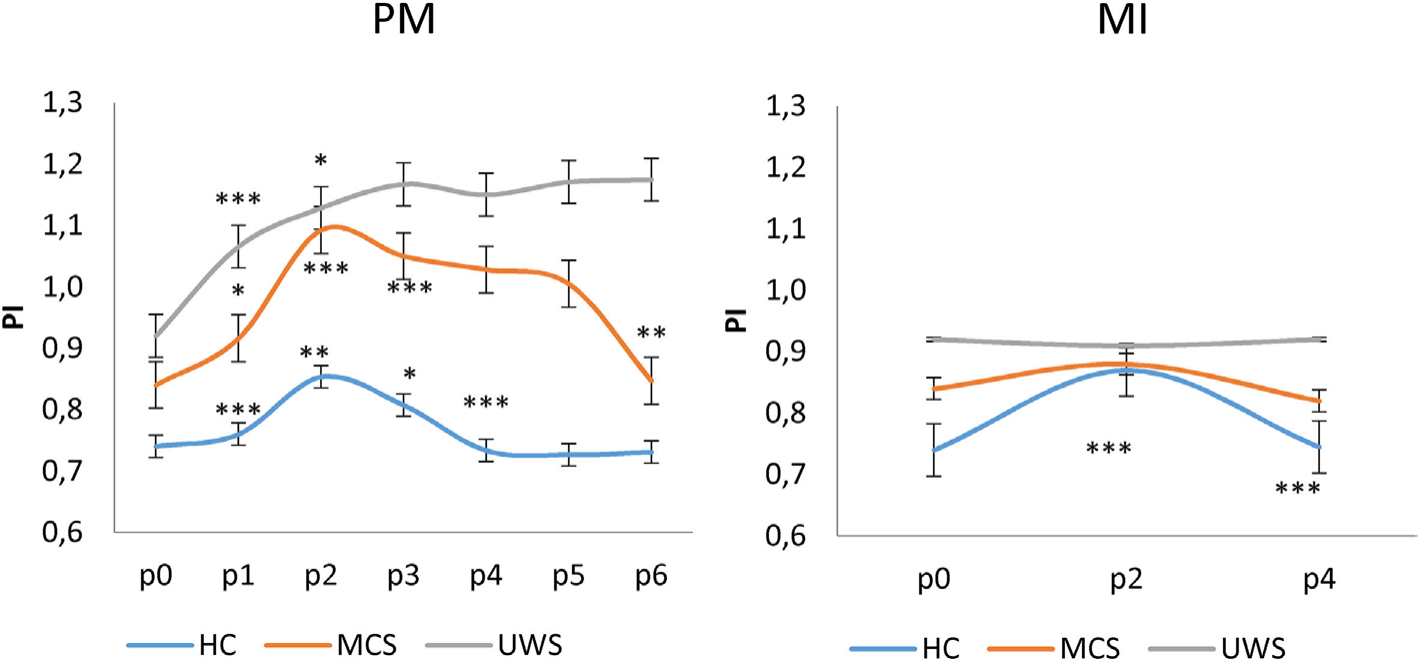
Shows mean (±SD; vertical error bar) pulsatility index (PI) values during passive movement (PM) and motor imagery (MI) tasks for each group [healthy controls (HC), minimally conscious state (MCS), and unresponsive wakefulness syndrome (UWS)] across the hand positions (p) explored. There was a significant difference at p0 (baseline) between the PI values of HCs and patients with disorders of consciousness (*p* < 0.001), and between patients with MCS and UWS (*p* = 0.01). PM induced a significantly different PI modulation at each *p* among the groups (*p* < 0.001). On the contrary, MI induced a significant increase of PI at p2 and decrease at p4 only in the HCs. *Refer to the significance of intragroup PI change at each *p* as compared to the previous one (Bonferroni corrected *p*-value, ***p < 0.001, **p = 0.001, *p < 0.0125).

**Figure 3:**
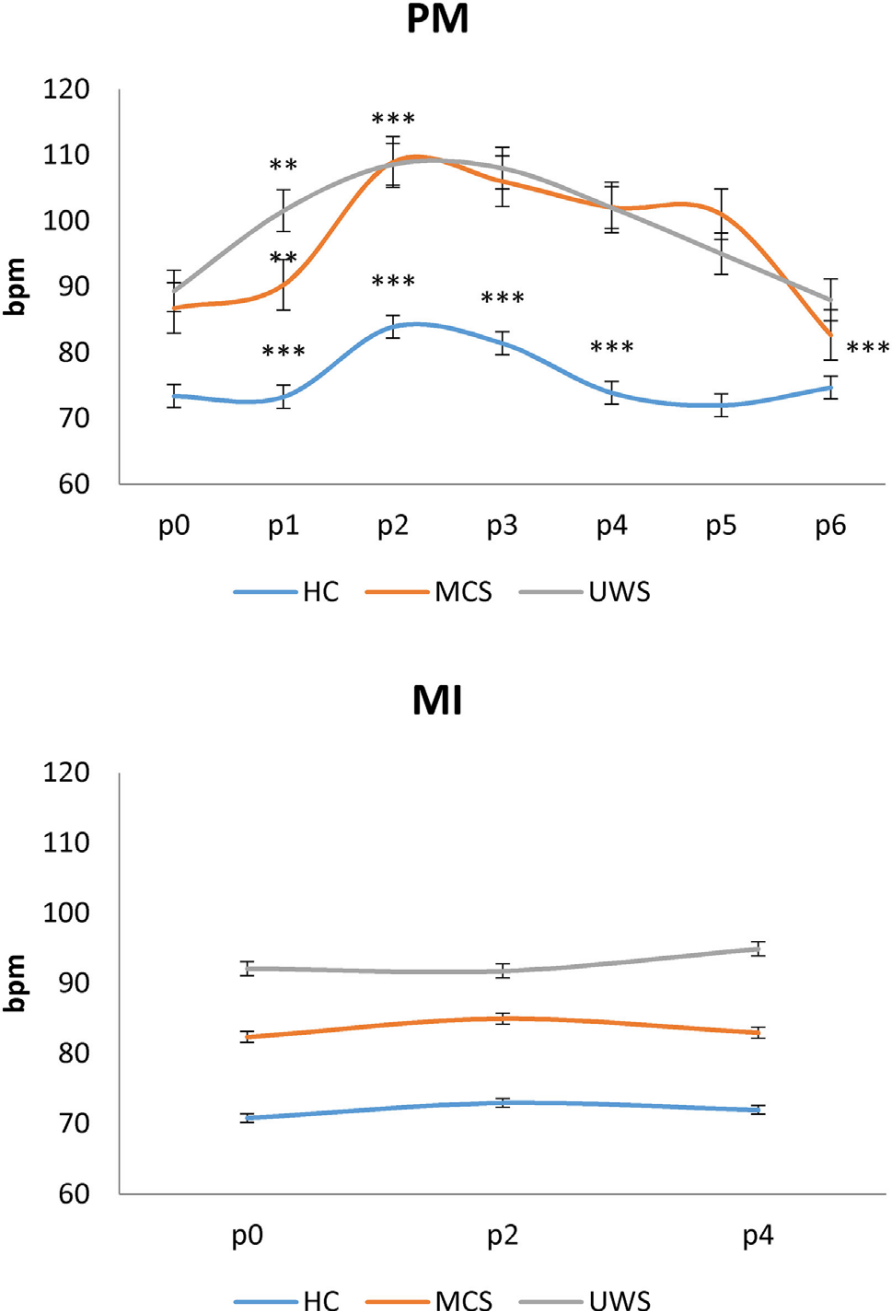
Shows mean (±SD; vertical error bar) heart rate values [in beats per minute (bpm)] during passive movement (PM) and motor imagery (MI) tasks for each group [healthy controls (HC), minimally conscious state (MCS), and unresponsive wakefulness syndrome (UWS)] across the hand positions (p) explored. There was a significant difference at p0 (baseline) between the bpm values of HCs and patients with disorders of consciousness (*p* < 0.001), and between patients with MCS and UWS (*p* = 0.01). PM induced a significantly different bpm modulation at each *p* among the groups (*p* < 0.001). On the contrary, MI did not induce any significant bpm change. *Refer to the significance of intragroup pulsatility index change at each *p* as compared to the previous one (Bonferroni corrected *p*-value, ***p < 0.001, **p = 0.001, *p < 0.0125).

**Figure 4:**
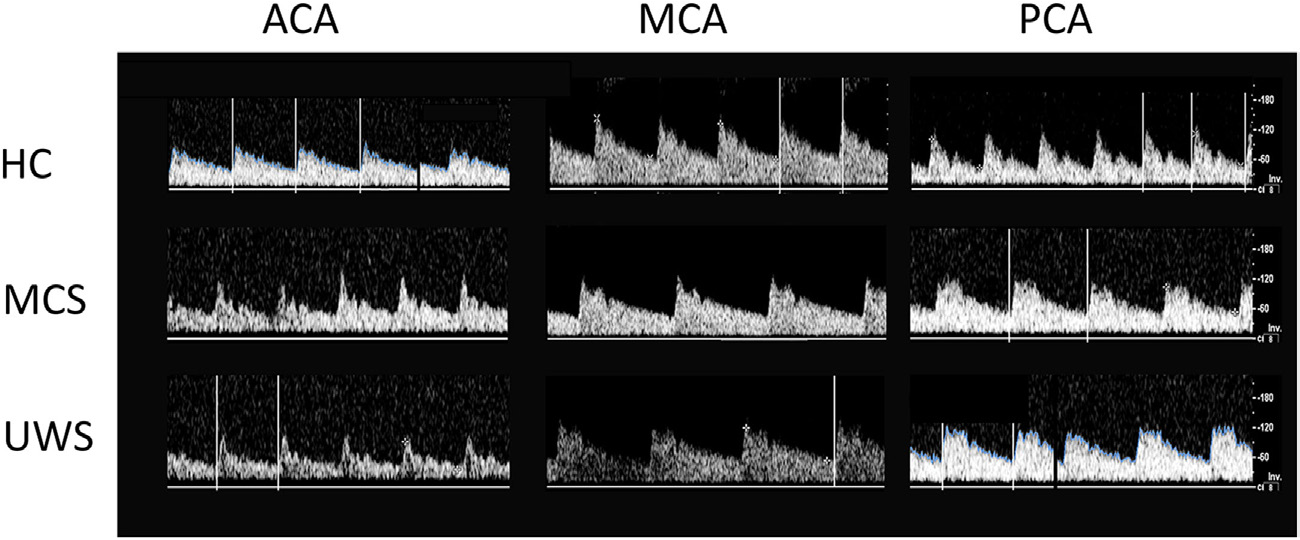
Shows the transcranial Doppler waveforms (on the right) from left middle (MCA), anterior (ACA), and posterior (PCA) cerebral arteries at rest in a representative subject for each group (HC, MCS, and UWS). Flow velocity (in centimeter per second, right vertical bar) are provided.

All the participants completed the experimental study without any side effect. Clinical, neurobehavioral, and functional study data were all concordant in the same patient for the diagnosis of either MCS or UWS. At baseline (p0_rest_), all the patients showed higher PI and HR values than HC individuals did (*p* < 0.001). Moreover, PI and HR values at rest were higher in patients with UWS than with MCS (*p* = 0.01), with no differences between MCS+ and MCS− (*p* > 0.1) (Figures 2 and 3; Table S1 in Supplementary Material). Nonetheless, some patients with MCS and UWS diverged from this trend, showing lower/higher PI and HR values (Table S1 in Supplementary Material). An example of CBF from the left anterior, middle, and posterior cerebral arteries at rest in one HC, one patient with MCS, and one patient with UWS is provided in Figure 4.

**Figure 5:**
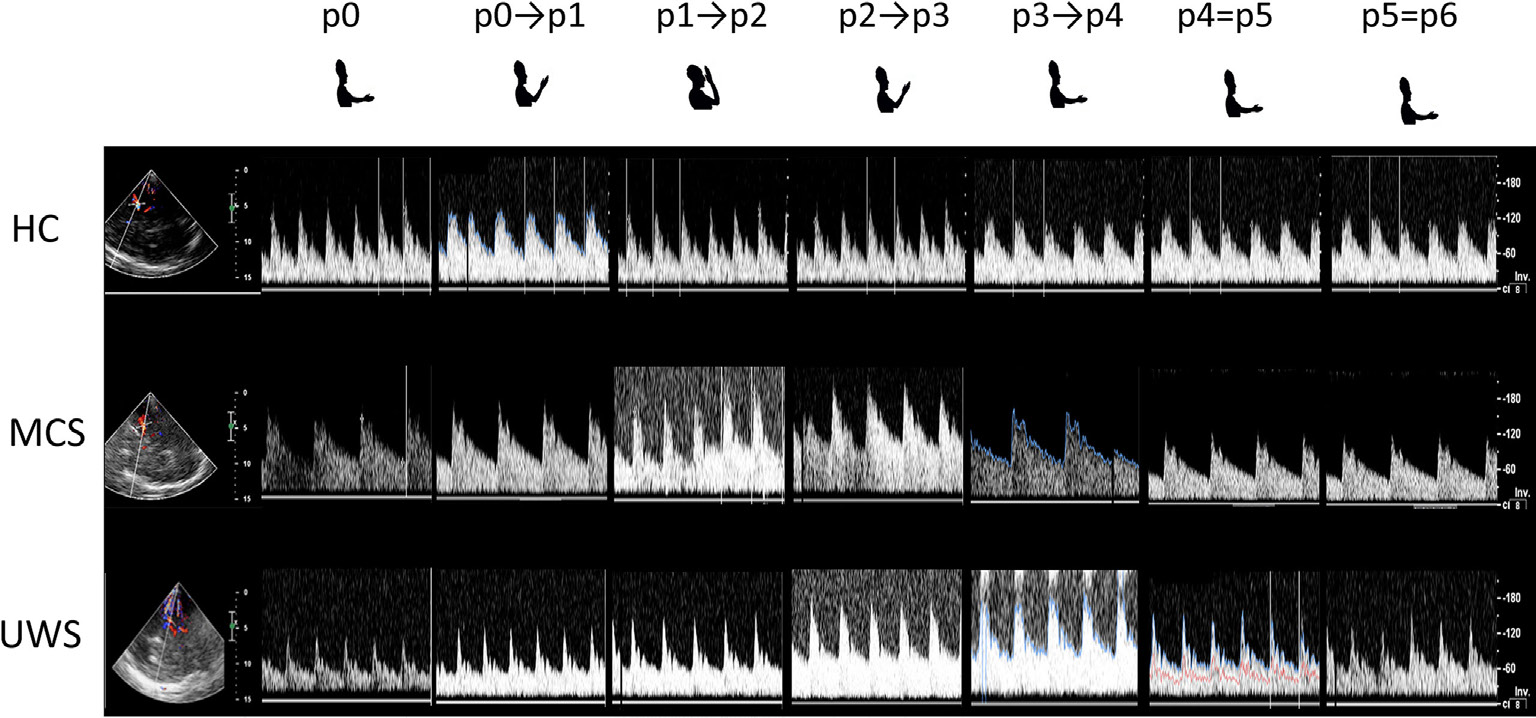
Shows the transcranial Doppler waveforms (on the right) with color Doppler (on the left) from left middle cerebral artery during passive mobilization across the six hand positions (p) explored in a representative subject for each group [healthy controls, minimally conscious state (MCS), and unresponsive wakefulness syndrome (UWS)]. Both insonation depth (left vertical bar) and flow velocity (centimeter per second, right vertical bar) are provided.

Motor imagery and passive movement tasks yielded significantly different effects on PI [*task* × *group* × *hand-position F*_(12,516)_ = 17, *p* < 0.001]. In detail, passive movement task determined significantly different changes of PI across the hand positions between the groups [*group* × *hand-position F*_(10,430)_ = 26, *p* < 0.001; *group F*_(2,86)_ = 24, *p* < 0.001; *hand-position F*_(5,215)_ = 21, *p* < 0.001]. In fact, all the HCs showed significant changes of PI values across the hand positions [*hand-position* effect *F*_(5,120)_ = 138, *p* < 0.001], according to the following schema: p0 < p1 < p2 > p3 > p4 ≈ p5 ≈ p6, with p3 ≈ p1 and p0 ≈ p4 ≈ p5 ≈ p6 (Figures 2 and 5; Table S1 in Supplementary Material; Table 2). All the patients with MCS showed significant changes of PI values across hand positions [*hand-position* effect *F*_(5,45)_ = 6.9, *p* < 0.001], with a pattern that differed from that observed in the HC: p0 < p1 < p2 > p3 ≈ p4 ≈ p5 > p6 (where p6 ≈ p0). There were no significant differences between patients with MCS+ and MCS− (*group effect p* = 0.2). Instead, all the patients with UWS showed a global increase of PI during the passive motor (PM) task, without the significant PI modulation according to the following pattern: p0 < p1 < p2 ≈ p3 ≈ p4 ≈ p5 ≈ p6 (*hand-position* effect *p* = 0.7). Therefore, the PI increased when the hand was moved toward the face in patients with DoC, as observed in the HCs. However, PI remained high when the hand was removed from the face, differently from what observed in the HCs. Further, the high levels of PI lasted up to 30 s after the end of the movement in patients with MCS (both MCS+ and MCS−) and 1 min in those with UWS. An example of CBF changes from the left middle cerebral artery across the positions (p) explored during the PM task in one HC, one patient with MCS, and one patient with UWS is provided in Figure 5. Intraindividual variability between the two trials was very low in all the participants (*p* < 0.005).

**Table 2:** *Post hoc t*-test concerning pulsatility index modulation at each hand position (p) as compared to the previous one (significant when *p*-value <0.0125), during the passive motor (PM) and motor imagery (MI) task in patients with minimally conscious state (MCS) and unresponsive wakefulness syndrome (UWS), and in healthy controls (HC). MI data of patients with UWS were not significant.

Group	PM	*t*-Value	*p*-Value
	Hand position		
HC	p0–p1	−8	<0.001
	p1–p2	−3.9	0.001
	p2–p3	2.8	0.01
	p3–p4	6.6	<0.001
	p4–p5		>0.1
	p5–p6		>0.1

MCS	p0–p1	−2.9	0.01
	p1–p2	−6.5	<0.001
	p2–p3	6.5	<0.001
	p3–p4		>0.1
	p4–p5		>0.1
	p5–p6	5.1	0.001

UWS	p0–p1	−7.2	<0.001
	p1–p2	−3.3	0.01
	p2–p3		>0.1
	p3–p4		>0.1
	p4–p5		>0.1
	p5–p6		>0.1

**Group**	**MI**	**t-Value**	**p-Value**
	**Hand position**		

HC	p0–p2	−8.2	<0.001
	p2–p4	6.5	<0.001

MCS	p0–p2	−2.1	0.07
	p2–p4	2.3	0.04

**Figure 6:**
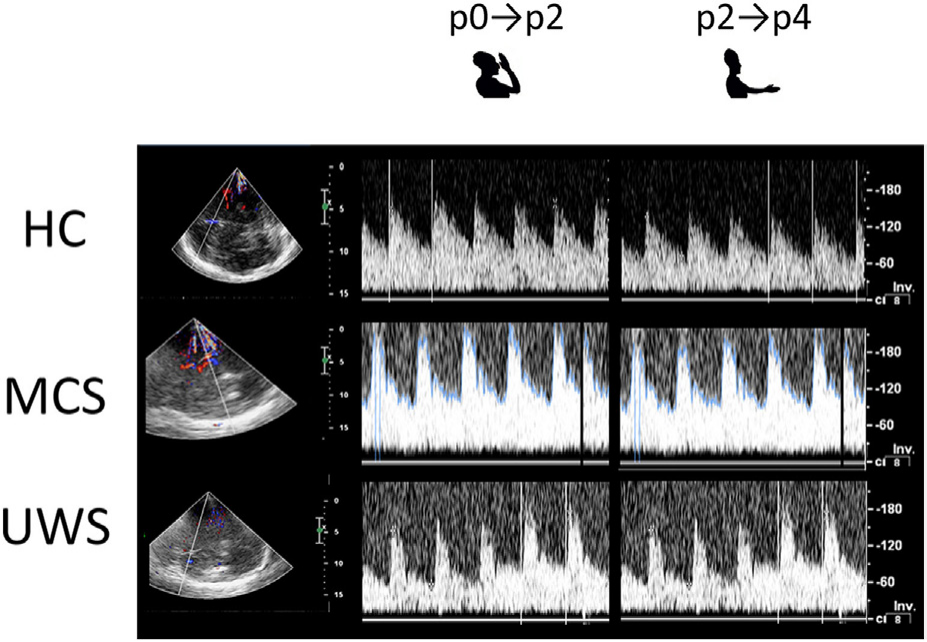
Shows the transcranial Doppler waveforms (on the right) with color Doppler (on the left) from left middle cerebral artery during motor imagery across the two hand positions (p) explored in a representative subject for each group [HC, minimally conscious state (MCS), and unresponsive wakefulness syndrome (UWS)]. Both insonation depth (left vertical bar) and flow velocity (cm/s, right vertical bar) are provided.

Motor imagery task significantly influenced the PI across hand positions between the groups [*group* × *hand-position F*_(10,430)_ = 10, *p* < 0.001; *group F*_(2,86)_ = 11, *p* < 0.001; *hand-position F*_(1,43)_ = 19, *p* < 0.001]. Indeed, all the HCs showed significant changes of PI values across the hand positions (p) according to the following schema: p0 < p2 > p4, with p0 ≈ p4 [*hand-position* effect *F*_(1,43)_ = 48, *p* < 0.001] (Figures 2 and 6; Table S1 in Supplementary Material; Table 2). Such a pattern was barely appreciable in the MCS group (*hand-position* effect *p* = 0.06), in which three patients out of four with MCS+ and one patient out of six with MCS− showed such pattern. Thus, we found no significant intra-MCS difference (*p* = 0.1), although the PI modulation in the three patients with MCS+ was greater than that observed in the patient with MCS−. On the other hand, PI modulation was absent in all the patients with UWS (*hand-position* effect *p* = 0.7). An example of CBF changes from the left middle cerebral artery across the positions (p) explored during the MI task in one HC, one patient with MCS, and one patient with UWS is provided in Figure 6. Intraindividual variability between the two trials was very low in all the participants (*p* < 0.005).

Heart rate during passive movement showed a pattern of increase from p0 to p2 and a decrease from p2 to p6 in each group [*hand-position F*_(5,215)_ = 9, *p* < 0.001; *group* × *hand-position p* = 0.1; *group p* = 0.9] as suggested by the significant *hand-position* effect in HC [*F*_(5,120)_ = 54, *p* < 0.001], patients with MCS [*F*_(5,45)_ = 4.9, *p* = 0.001] and with UWS [*F*_(5,50)_ = 6.6, *p* < 0.001] (Figure 3; Table 3). On the contrary, HR did not significantly change during MI in any group (all interactions and effects *p* > 0.1) (Figure 3).

**Table 3:** *Post hoc t*-test concerning heart rate (HR) modulation at each hand position (p) as compared to the previous one (significant when *p*-value < 0.0125), during the passive motor task in patients with minimally conscious state (MCS) and unresponsive wakefulness syndrome (UWS), and in healthy controls (HC). HR data of motor imagery were not significant.

Group	Hand position	*t*-Value	*p*-Value
HC	p0–p1	−7.4	<0.001
	p1–p2	−5.5	<0.001
	p2–p3	8.2	<0.001
	p3–p4	14	<0.001
	p4–p5		>0.1
	p5–p6		>0.1

MCS	p0–p1	−3.3	0.008
	p1–p2	−2.9	0.02
	p2–p3	2.4	0.04
	p3–p4		>0.1
	p4–p5		>0.1
	p5–p6	4.2	0.001

UWS	p0–p1	−3.5	0.006
	p1–p2	−5.1	<0.001
	p2–p3		>0.1
	p3–p4		>0.1
	p4–p5		>0.1
	p5–p6		>0.1

In the control experiment (10 subjects), in which the experimenter’s hand entered the PPS-face while the subject remained still, we did not document any significant PI changes across the entire range of movement.

We found a significant correlation between the best CRS-R score and the PI modulation during passive movement (*r* = 0.623, *p* = 0.002). There were no significant effects of clinical-demographic characteristics (age, β = 0.01, *p* = 0.5; gender, β = 0.01, *p* = 0.4; disease duration, β = 0.26, *p* = 0.5; and treatment, β = 0.13, *p* = 0.6) on PI changes across motor tasks, as well as an effect of blood pressure and HR on PI (all *p* > 0.1). Also, brain etiology (β = 0.01, *p* = 0.4) and risk factors (β = 0.01, *p* = 0.7) did not influence the dependent variable PI.

**Figure 7:**
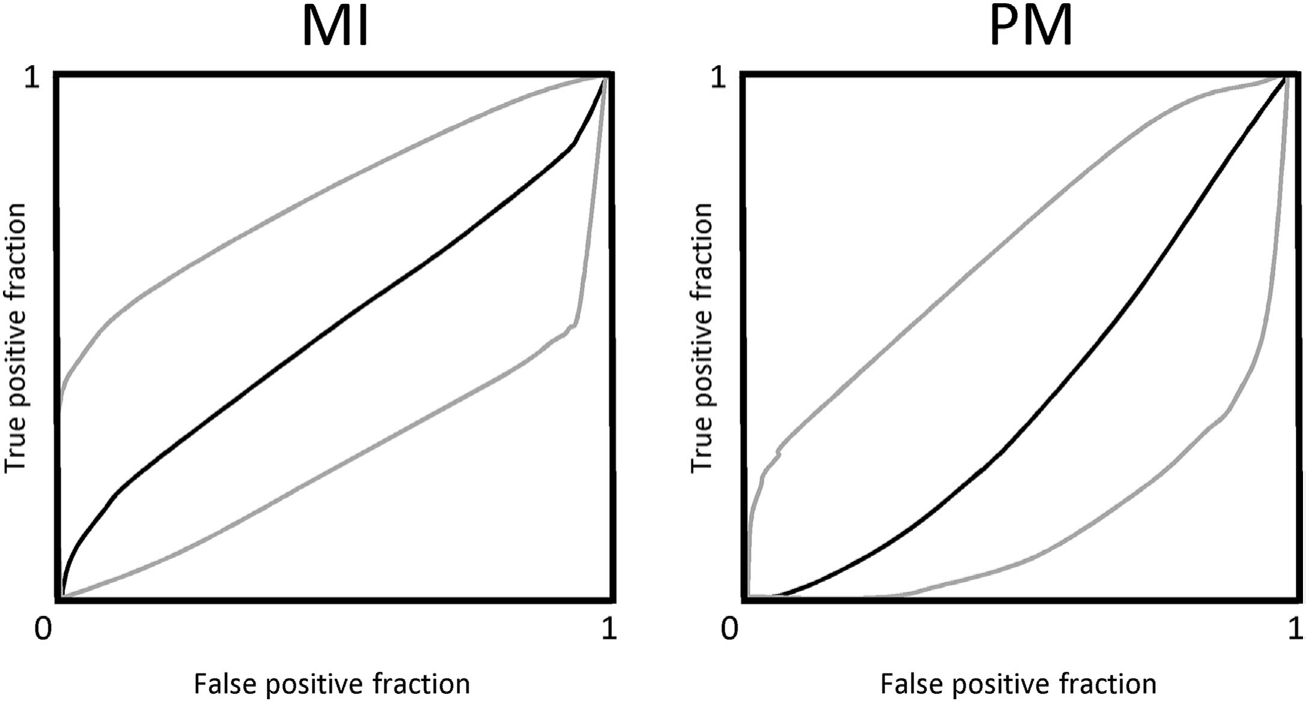
The power of motor task-induced cerebral blood flow velocity modulation across the hand-position employed in differentiating patients with disorders of consciousness is shown by the area under the receiver operating characteristic curve (AUC) for motor imagery (MI) and passive mobilization tasks.

Concerning the sensitivity analysis, we employed a linear regression model; SRCs were considered as direct measures of sensitivity. We found that the PI changes from p0 to p2 and from p2 to p4 (which were the most significant intervals), were the most predictive values for DOC diagnosis during the passive movement task (SRC = 0.05, *p* = 0.01). Finally, the ROC analysis with PI value and CRS-R score as factors showed that the diagnostic accuracy of the overall PI magnitude modulation during passive movement was good (AUC = 0.6), with a sensitivity and specificity concerning DoC category of 100%. In contrast, MI task-induced PI magnitude modulation was poorly associated with CRS-R score (AUC = 0.3) (Figure 7), with a sensitivity of 40% and a specificity of 100% concerning DoC category.

## Discussion

To the best of our knowledge, this is the first attempt to characterize PPS in patients with DoC. Our data suggest that fTCD evaluation of PPS functions may be useful to corroborate the differential diagnosis of patients with DoC. In fact, all the patients with DoC showed an increased baseline vascular reactivity (i.e., higher PI and HR values) as compared to HC individuals, in keeping with the uncoupling of CBF and cerebral metabolic rate arising from reduced cerebral glucose consumption and oxygen uptake after extensive brain injury (37–39). Even though the patients with UWS showed higher PI and HR values at baseline than those with MCS, some patients diverged from their group trend, in that they had lower/higher PI and HR values. Moreover, CRS-R scores did not correlate with baseline PI and HR values. On the other hand, the assessment of CBFV during the PM task targeting the PPS allowed differentiating between patients with MCS and UWS. Even though the PI increased when the hand was moved toward the face in patients with DoC, it remained high when the hand was removed from the face and up to 30 s after the end of the movement in the patients with MCS (both MCS+ and MCS−) and 1 min in those with UWS, thus differentiating between patients with MCS and UWS. This pattern of CBFV changes is in keeping with the protective role played by PPS toward the potential threats approaching body parts. This role is reflected by a more vigorous defensive reactions elicited when stimuli are located inside rather than outside the PPS (18, 19, 21, 22). The lack of an early normalization of CBFV in patients with DoC may be related to a dysfunction in the regulation of temporized brain activity or in the replenishing of the metabolite levels after increased brain activity (40, 41). The strong impairment of brain metabolism and the tonic cortical and subcortical dis-excitability may account for the delay in CBFV normalization observed in patients with DoC (42–44). Additionally, a dysfunctional neurovascular coupling, i.e., the close spatial and temporal relationships between the neural activity and CBF, also accounts for the lack of CBFV normalization following a task (39, 45).

Noteworthy, our findings may agree with the reported correlation between the behavioral responsiveness of patients with DoC and the degree of cortico-subcortical connectivity breakdown and subcortical hyper-connectivity (46). In fact, the magnitude and extent of PI modulation during PM task (but not the baseline values) were correlated with the CRS-R scoring, i.e., the lower and more reflex the behavioral responsiveness, the higher and longer the PI increase. Therefore, CBFV regulation may reflect the partial preservation of top-down modulation processes from higher-order cortical areas to sensory-motor integration networks related to the PPS (16, 20, 21, 24, 47, 48), in patients with MCS but not in those with UWS.

Interestingly, four patients with MCS (three with MCS+ and one with MCS−) showed a barely appreciable PI modulation during the MI task. Studies testing MI in patients with MCS disclosed a high rate of false negative, i.e., a patient can have some motor function but does not demonstrate brain activity when asked to imagine a task (49–51). According to these issues, PI modulation during MI does not seem a very reliable method for differentiating the MCS status, but it may only furnish information about the differential diagnosis between MCS and UWS. The lack of sensitivity of PI modulation during MI task may depend on the nature of the brain processes related to stimuli approaching PPS, i.e., protective response, which is not the case of MI. Moreover, it is likely that that passive movement induces a neurovascular activation that is not detectable during MI, which instead represents an internally triggered event (52). Finally, the simplicity of the movement required, in comparison to the more complex MI tasks formerly employed (i.e., playing tennis or in-house navigation), could have yielded non-specific cerebral blood flow responses that are independent of the motor task.

Heart rate showed a common pattern of modulation during passive movement task without any correlation to PI and CRS-R, whereas it did not change during MI. Thus, hemodynamic factors like HR seem to not bias PI while performing such motor tasks.

### Limitations

The small sample enrolled represents a main limitation of our study. Moreover, none of the patients with MCS had borderline CRS-R scores between MCS and UWS (i.e., in the range of 6–8), and only 2 patients with MCS had a score of 9. This issue might have magnified the observed fTCD differences between patients with MCS and UWS. Also, we did not find fTCD differences between MCS+ and MCS−, which may depend on the CRS-R scores the patients with MCS− reported (up to 14, which makes them in the higher boundaries of MCS−). If we had available patients with MCS− with CRS-R score in the range of 6–8, we could have differentiated patients with MCS+ and MCS−. Therefore, future studies with a larger number of patients, a more varied CRS-R score range, and long-term outcomes, should be undertaken to confirm the possible use of fTCD as a complementary diagnostic tool. Also, fMRI or PET-scans should be used to confirm our findings, as fTCD could represent an approach that indicates the subject candidates to undergo advanced and sophisticated neuroimaging paradigms.

Functional transcranial doppler measures CBFV rather than absolute cerebral blood flow. An estimation of the latter can be made if the diameter of the insonated vessel remains constant (25, 26), but there is not sufficient data to demonstrate this issue in our work. fTCD has an interesting temporal resolution, but the spatial resolution is unfortunately low so that we cannot be precise on spatialized cerebral hemodynamics.

We did not measure right arm electromyography to exclude possible voluntary muscular activity during the passive movement, even though previous studies have ruled out significant biasing effects on CBFV of muscle activation during a passive movement task (25, 26).

A possible contribution from peripheral covariates (including beat-to-beat arterial blood pressure, HR, PaCO_2_, breath-by-breath end-tidal CO_2_, and the neural activation stimulus represented by the go-signal) could lead to the inaccurate assessment of CBFV, particularly during MI (25, 26). About that, blood pressure and HR were monitored and did not significantly correlate with PI values. However, a larger assessment of such peripheral covariates will be necessary. In fact, it would be important to specifically identify the presence of a dysautonomic syndrome and of other alteration of the mechanisms of cerebral vascular autoregulation. However, the blood pressure and HR (which were monitored during the experimental session) changed according to a common waxing–waning pattern. Moreover, brain etiology and the presence of risk factors (both added to the multivariate analysis) did not significantly influence the PI changes. Nonetheless, larger samples should be investigated to define these issues better.

Finally, one could be concerned that PI modification may be simply related to hand movement protocol rather than to PPS violation response. However, the condition, in which the experimenter’s hand entered the PPS while the upper limb of the participant was not moved, showed no significant PI changes. Therefore, it is reasonable that PI changes are related to PPS violation rather than to the hand movement.

### Conclusions

Functional transcranial Doppler could be a promising, quick, and easy tool for the bedside differential diagnosis between patients with MCS and UWS. Indeed, fTCD helps to identify CBF changes that are related to top-down modulation processes from higher-order cortical areas to sensory-motor integration networks related to the PPS, when using passive movement tasks. Despite the small sample size and the simplicity of the methodology as compared to those more advanced (53, 54) our approach may also allow the clinician to identify the candidates for carrying out advanced and sophisticated neuroimaging tools when they are not readily available.

## Ethics Statement

All procedures performed in studies involving human participants were in accordance with the ethical standards of the institutional and/or national research committee and with the 1964 Helsinki declaration and its later amendments or comparable ethical standards. Our Local Ethic Committee approved the study.

## Informed Consent

Healthy subject and the legal surrogates of patients with Disorder of Consciousness gave their written informed consent to study participation.

## Author Contributions

AN, RC, and PB: substantial contributions to the conception and design of the work, and interpretation of data; revising the work critically for important intellectual content; final approval of the version to be published; agreement to be accountable for all aspects of the work in ensuring that questions related to the accuracy or integrity of any part of the work are appropriately investigated and resolved. AC, SP, AB, and RL: acquisition and analysis of data; drafting the work; final approval of the version to be published; and agreement to be accountable for all aspects of the work in ensuring that questions related to the accuracy or integrity of any part of the work are appropriately investigated and resolved.

## Conflict of Interest Statement

The authors declare that the research was conducted in the absence of any commercial or financial relationships that could be construed as a potential conflict of interest. The reviewer AT and handling editor declared their shared affiliation.

## Supplementary Material

Table S1 Shows individual pulsatility index (PI) values during passive mobilization and motor imagery (MI) for each group [healthy controls (HC), minimally conscious state (MCS)±, and unresponsive wakefulness syndrome (UWS), with their coma recovery scale-revised score in parentheses] across the six positions explored and the two imagined movements (p). The patients with MCS whose pulsatility index values differed from those of the other individuals belonging to the same subgroup (MCS+ or MCS−) are highlighted in red.
